# MRI-Only Radiotherapy Planning for Nasopharyngeal Carcinoma Using Deep Learning

**DOI:** 10.3389/fonc.2021.713617

**Published:** 2021-09-08

**Authors:** Xiangyu Ma, Xinyuan Chen, Jingwen Li, Yu Wang, Kuo Men, Jianrong Dai

**Affiliations:** ^1^National Cancer Center/National Clinical Research Center for Cancer/Cancer Hospital, Chinese Academy of Medical Sciences and Peking Union Medical College, Beijing, China; ^2^Cloud Computing and Big Date Research Institute, China Academy of Information and Communications Technology, Beijing, China

**Keywords:** nasopharyngeal carcinoma, radiotherapy, MRI-only planning, pseudo CT, deep learning, dosimetric evaluation

## Abstract

**Background:**

Radical radiotherapy is the main treatment modality for early and locally advanced nasopharyngeal carcinoma (NPC). Magnetic resonance imaging (MRI) has the advantages of no ionizing radiation and high soft-tissue resolution compared to computed tomography (CT), but it does not provide electron density (ED) information for radiotherapy planning. Therefore, in this study, we developed a pseudo-CT (pCT) generation method to provide necessary ED information for MRI-only planning in NPC radiotherapy.

**Methods:**

Twenty patients with early-stage NPC who received radiotherapy in our hospital were investigated. First, 1433 sets of paired T1 weighted magnetic resonance (MR) simulation images and CT simulation images were rigidly registered and preprocessed. A 16-layer U-Net was used to train the pCT generative model and a “pix2pix” generative adversarial network (GAN) was also trained to compare with the pure U-Net regrading pCT quality. Second, the contours of all target volumes and organs at risk in the original CT were transferred to the pCT for planning, and the beams were copied back to the original CT for reference dose calculation. Finally, the dose distribution calculated on the pCT was compared with the reference dose distribution through gamma analysis and dose-volume indices.

**Results:**

The average time for pCT generation for each patient was 7.90 ± 0.47 seconds. The average mean (absolute) error was −9.3 ± 16.9 HU (102.6 ± 11.4 HU), and the mean-root-square error was 209.8 ± 22.6 HU. There was no significant difference between the pCT quality of pix2pix GAN and that of pure U-Net (p > 0.05). The dose distribution on the pCT was highly consistent with that on the original CT. The mean gamma pass rate (2 mm/3%, 10% low dose threshold) was 99.1% ± 0.3%, and the mean absolute difference of nasopharyngeal PGTV D_99%_ and PTV V_95%_ were 0.4% ± 0.2% and 0.1% ± 0.1%.

**Conclusion:**

The proposed deep learning model can accurately predict CT from MRI, and the generated pCT can be employed in precise dose calculations. It is of great significance to realize MRI-only planning in NPC radiotherapy, which can improve structure delineation and considerably reduce additional imaging dose, especially when an MR-guided linear accelerator is adopted for treatment.

## Introduction

Nasopharyngeal carcinoma (NPC) is the most common malignant tumor in the head and neck (HN), especially in southern China and Southeast Asia. Radical radiotherapy (RT) is the main treatment modality for early or locally advanced NPC, and computed tomography (CT) is necessary for patient positioning and RT planning, since it provides electron density (ED) information for dose calculation. Magnetic resonance imaging (MRI) has the advantages of high soft-tissue resolution and no additional imaging dose compared to CT. With the development of the MR-guided linear accelerator (MR-linac), an *MRI-only* RT-planning workflow is desirable. However, MRI does not provide ED information, which hinders its application in RT planning. Therefore, there is a need for a reliable and effective method to predict ED information based on MR images.

Currently, this issue is addressed using three main methods. The primary one is to simply segment soft tissue and bone (1, 2) and assign the densities of water and bone to them, respectively. However, it is difficult to distinguish between bone and air in MRI.

The second is the atlas-based pseudo-CT (pCT) generation. It requires a deformable registration from an MRI atlas to the patient MRI to obtain a special transformation, which is then applied to a paired CT atlas to generate pCT images (3–8). However, when the patient’s MRI is quite different from the image in the atlas library, and there is a special anatomical structure (such as a large tumor or surgical cavity), leading to deformation registration errors, which affect the accuracy of pCT.

The third is the voxel-wise pCT generation (9–12). By establishing a voxel-wise pCT generation model, point-by-point prediction is performed. This method prevents manual or semi-manual segmentation of soft tissue and bone, and it is not sensitive to abnormal anatomical structures. Earlier studies employed machine learning methods, such as cluster analysis, Gaussian regression, and principal component analysis, to establish such a generative model. However, some of them still need manual or semi-automatic delineation of bone and air cavity, and the prediction accuracy still needs to be improved.

Recently, convolutional neural network (CNN) and its derivate deep learning models have been widely used for cross-modality image generation owing to their ability to automatically extract multilevel features of data. At present, most studies on MR-pCT generation focus on brain and prostate RT (11, 13–16), and promising accuracy has been achieved. However, there is a need for further studies to develop and verify deep learning based pCT generative models for treatment sites with more intertwined air cavities and bony structures, such as HN. A previous study (17) used U-Net and T2-weighted (T2w) MRI to generate HN pCT for NPC and reported a promising image quality. In this study, we adopted U-Net but with T1w MRI, another routine clinical MRI modality, to train an MR-pCT generative model for NPC. We not only evaluated the CT number prediction accuracy but also systematically analyzed the dosimetric difference between the obtained pCT and the corresponding original CT with the same beam layout. Besides, we compared the performance of the generative adversarial network (GAN), another popular deep learning network, with U-Net for pCT generation.

## Materials and Methods

### Image Collection

The image data in this study were obtained from 20 patients with NPC who received RT in our hospital from September 2017 to April 2018. All data are retrospective and nonidentifiable so that the institutional ethics review and written consent are exempted.

Before treatment, all patients underwent CT and MR simulation scanning in our department within very close time and with the same fixing devices for each patient. CT scanning was performed using a CT simulator (SOMATOM Definition AS 40, Brilliance CT big bore, Philips) with the acquisition parameters (voltage: 120 kV; exposure: 240 mAs; pitch: 0.94; image size: 512 × 512; pixel spacing: 0.96 mm; slice thickness: 3.0 mm). MRI scanning was performed using a 3.0-T MR simulator (Discovery MR750w, GE Healthcare) with a 6-channel split head coil and T1-FSE sequence with the acquisition parameters (repetition time (TR): 834 ms; echo time (TE): 7.96 ms; flip angle: 111°; image size: 512 × 512; pixel spacing: 0.55 mm; and slice thickness: 3 mm). All patients were fixed with head-neck-shoulder thermoplastic film. The upper boundary of the scanning range is half of the frontal sinus, and the lower boundary extends to the supraclavicular region.

### Data Preprocessing

Due to the design and characteristics of the coil, the signal intensity distribution of the same tissue might be uneven. We used an N3 algorithm to calibrate the bias field and performed gray value normalization and histogram matching of the MR images. Then, the MR and CT images were rigidly registered, and outlines were drawn on the aligned CT and MR images, respectively, using the thresholding segmentation algorithm. The overlap of the two outlines was used to generate a mask, and the density outside the mask was set equal to that of air.

### Deep Learning Architecture for MR-pCT Generation

Two deep learning models, CNN and conditional GAN (cGAN), were adopted in this study for comparison. For both models, we used several data enhancement techniques, including random clipping and flipping, to expand the number of data. The Adam method was used to optimize the loss function. The initial learning rate was set to 0.0002, and the maximum number of iterations was set to 40000. The network training and test were based on the Tensorflow platform and NVIDIA Tesla K80 GPU.

**Figure 1:**
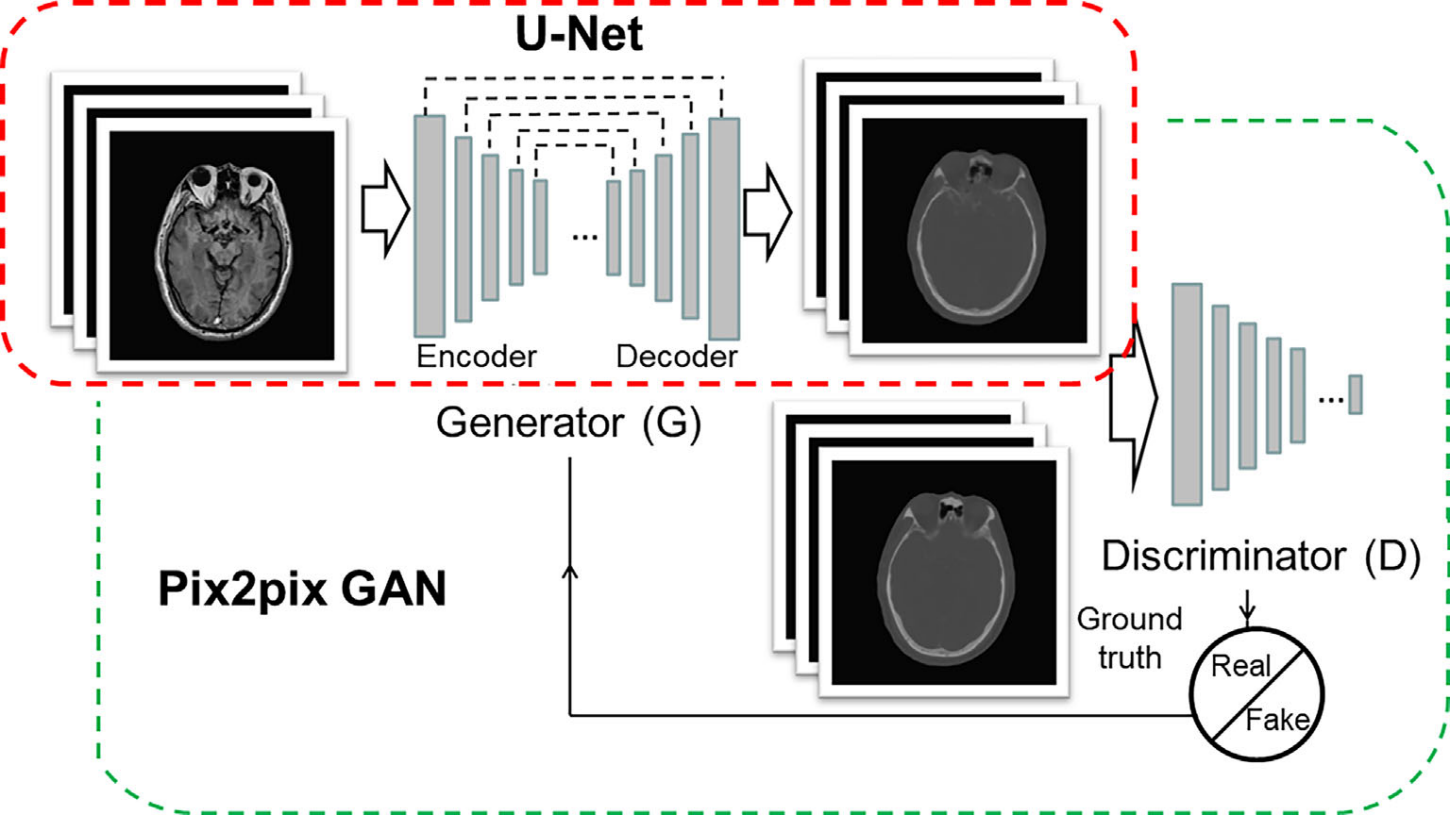
Architectures of U-Net (red box) and pix2pix GAN (green box). The U-Net is composed of an encoder and a decoder, and each of them has eight mosaic layers, which are detailed in **Table 1**.

The architecture of the CNN model was 16-layer U-Net, which was developed for MR-pCT generation (**Figure 1**, *red box*). As shown in **Table 1**, the modules of convolution-batch normalization and rectified linear units (ReLU) were used for the encoder–decoder network. The kernel size was 4 × 4 for the convolutional and deconvolutional layers. Skip connections were added between each mirrored encoder and decoder layers for better recovery of image details.

**Table 1:** Sixteen-layer U-Net architecture.

Encoder	Decoder
Conv 1 + BN + ReLU(512 × 512 × 64)	De_Conv 9 + BN + ReLU(4 × 4 × 512)
Conv 2 + BN + ReLU(256 × 256 × 128)	De_Conv 10 + BN + ReLU(8 × 8 × 512)
Conv 3 + BN + ReLU(128 × 128 × 256)	De_Conv 11 + BN + ReLU(16 × 16 × 512)
Conv 4 + BN + ReLU(64 × 64 × 512)	De_Conv 12 + BN + ReLU(32 × 32 × 512)
Conv 5 + BN + ReLU(32 × 32 × 512)	De_Conv 13 + BN + ReLU(64 × 64 × 256)
Conv 6 + BN + ReLU(16 × 16 × 512)	De_Conv 14+ BN + ReLU(128 × 128 × 128)
Conv 7 + BN + ReLU(8 × 8 × 512)	De_Conv 15 + BN + ReLU(256 × 256× 64)
Conv 8 + BN + ReLU(4 × 4 × 512)	De_Conv 16 + BN + ReLU(512 × 512 × 1)

The encoder input and decoder output image sizes are both 512 × 512. Conv, convolution; De_Conv, deconvolution; BN, Batch normalization; ReLU, rectified linear units.

The architecture of cGAN, as shown in **Figure 1** *(green box)*, was the “pix2pix” model, which used paired MR and CT images as input and ground truth, respectively. It learned a loss that uses a discriminator to determine if an output image is real or fake while simultaneously training a generator to minimize the loss. GAN is supposed to have the ability to overcome problems such as image blurring. For better comparison with the performance of U-Net, the generator part adopted the same U-Net architecture as aforementioned. The patch size of the discriminator was set to 70 × 70. We adopted “cGAN + L1” as a loss function, as suggested in (18). It comprises a standard cGAN loss function and a weighted L1 distance term.

To get a reliable and stable model based on a small sample size, a 10-fold cross-validation method was used to train the pCT generation model. Through cross-validation, optimal model parameters were determined and then used to generate pCT images for the 20 patients. Then, voxel-wise Hounsfield units (HU) comparison was performed between the pCT and the original CT for each patient, considering the mean error (ME), mean absolute error (MAE), and root-mean-square error (RMSE) (Equations 1–3).

(1) $$MAE=\frac{1}{N}\sum_{i=1}^{N}\left|p(i)-g(i)\right|$$

(2) $$ME=\frac{1}{N}\sum_{i=1}^{N}\left(p(i)-g(i)\right)$$

(3) $$RMSE=\sqrt{\sum_{i=1}^{N}\frac{p{\left(i\right)}^{2}-g{\left(i\right)}^{2}}{N}}$$

where *N* is the total number of voxels of interest, *p(i)* the value of the *i-th* voxel in the pseudo-CT, and *g(i)* the corresponding voxel value in the ground truth (original) CT.

### Dosimetric Evaluation

For each patient, contours of target volumes and organs at risk (OARs) were transferred from the original planning CT by rigid fusion to the corresponding pCT. RT plans were first designed with a Pinnacle treatment planning system (Philips) based on the pCT, and the beams were then copied to the original CT with the same isocenter for the ground truth dose calculation. Gamma analysis was performed using Sun Nuclear Patient (SNC Patient) software with a 2 mm/3% (global mode, 10% low dose threshold) criterion to compare dose distributions of pCT and original CT. The gamma criterion for the calculation is inconsistent in the literature, ranging from 1 mm/1% to 3 mm/3% (14–16). Hence, we used an intermediate value herein. For each patient, 10 slices with a 10-mm interval near the image central slice were selected for 2D gamma analysis, and the mean gamma pass rate of the slices was calculated for dosimetry consistency assessment. Besides, a dose-volume histogram (DVH) comparison was performed to evaluate the accuracy of the clinically concerned dosimetry metrics of PTVs and OARs.

## Results

### Performance Comparison Between U-Net and pix2pix GAN

**Figure 2:**
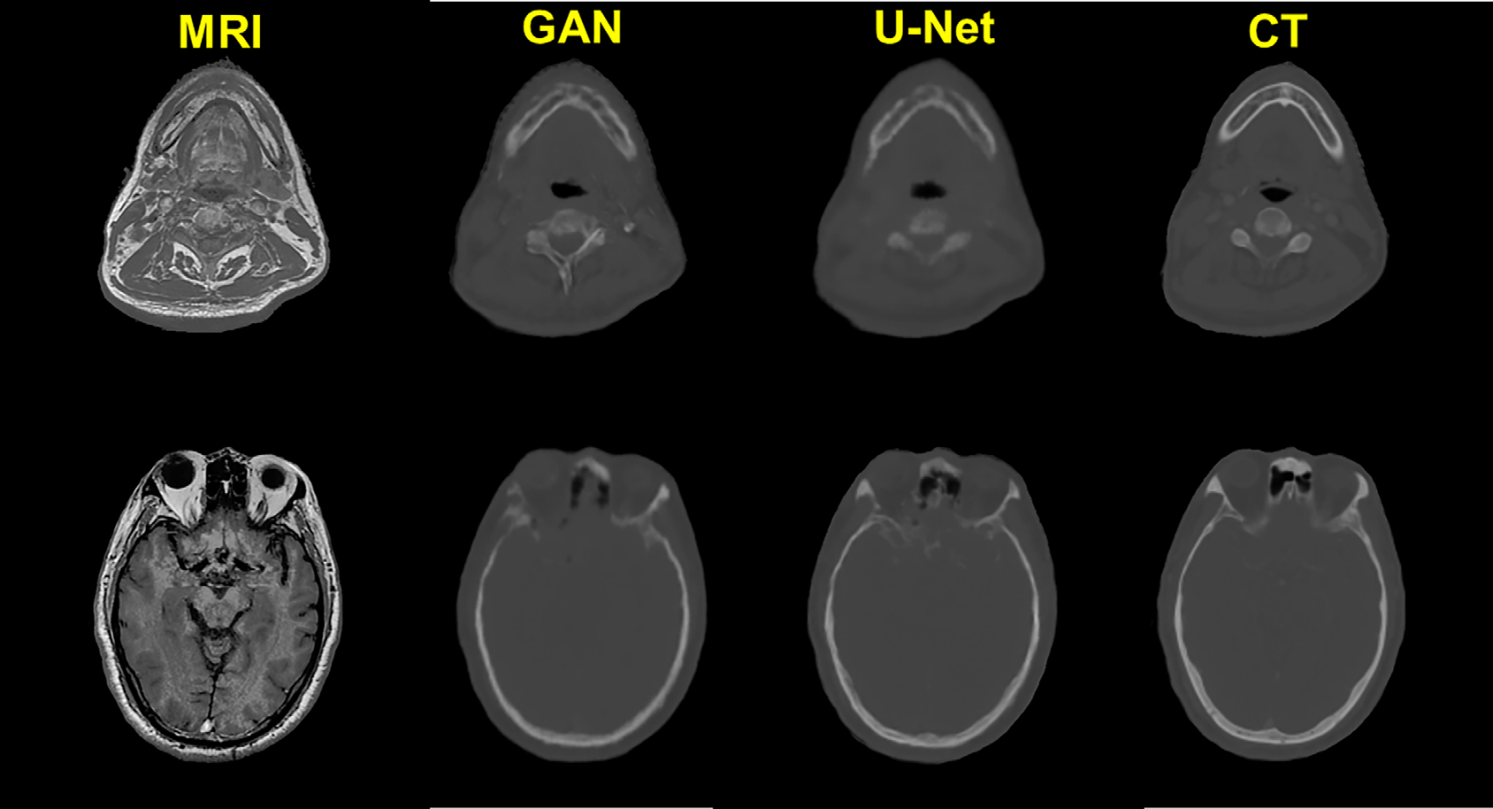
Comparison of the prediction results of U-Net and pix2pix GAN on two exemplary slices.

**Figure 3:**
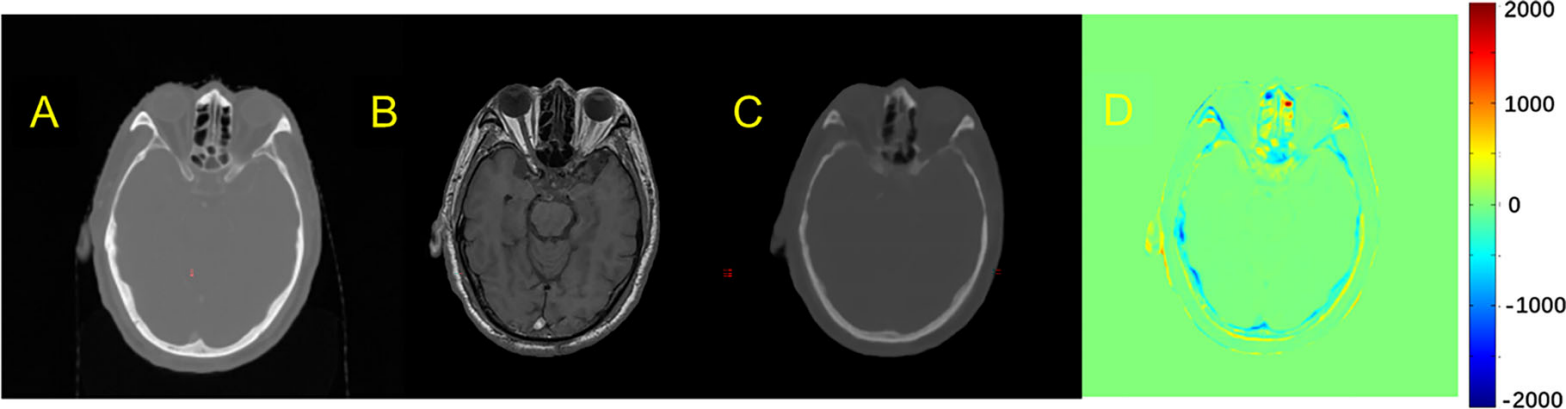
Comparison example of pCT and original CT: **(A)** Original CT images; **(B)** T1-weighted MR images; **(C)** Predicted pCT images; **(D)** Difference between the real CT and predicted pCT values, where MAE is 73.1 HU.

The quality of pCT generated by U-Net and pix2pix GAN has no statistical difference (paired t-test, p > 0.05) in terms of ME, MAE, and RMSE (**Table 2**), whereas the performance of U-Net was slightly better than that of pix2pix GAN. A visual comparison of the pCTs generated by the two types of networks is shown in **Figure 2**, and a spatial discrepancy map for the U-Net is shown in **Figure 3**. Since there was no significant difference between the performance of U-Net and pix2pix GAN in this task, we adopted the simpler-structured U-Net for the further dosimetric comparison.

**Table 2:** Prediction performance comparison of U-Net and pix2pix GAN.

Quality metrics	U-Net	GAN	*p-value
Average ME (HU)	−9.3 ± 16.9	−8.7 ± 17.3	0.325
Average MAE (HU)	102.6 ± 11.4	104.2 ± 12.5	0.051
Average RMSE (HU)	209.8 ± 22.6	213.2 ± 24.1	0.067

*Paired t-test.

### Dosimetric Consistency Between Real CT and pCT-Based RT Planning

**Figure 4:**
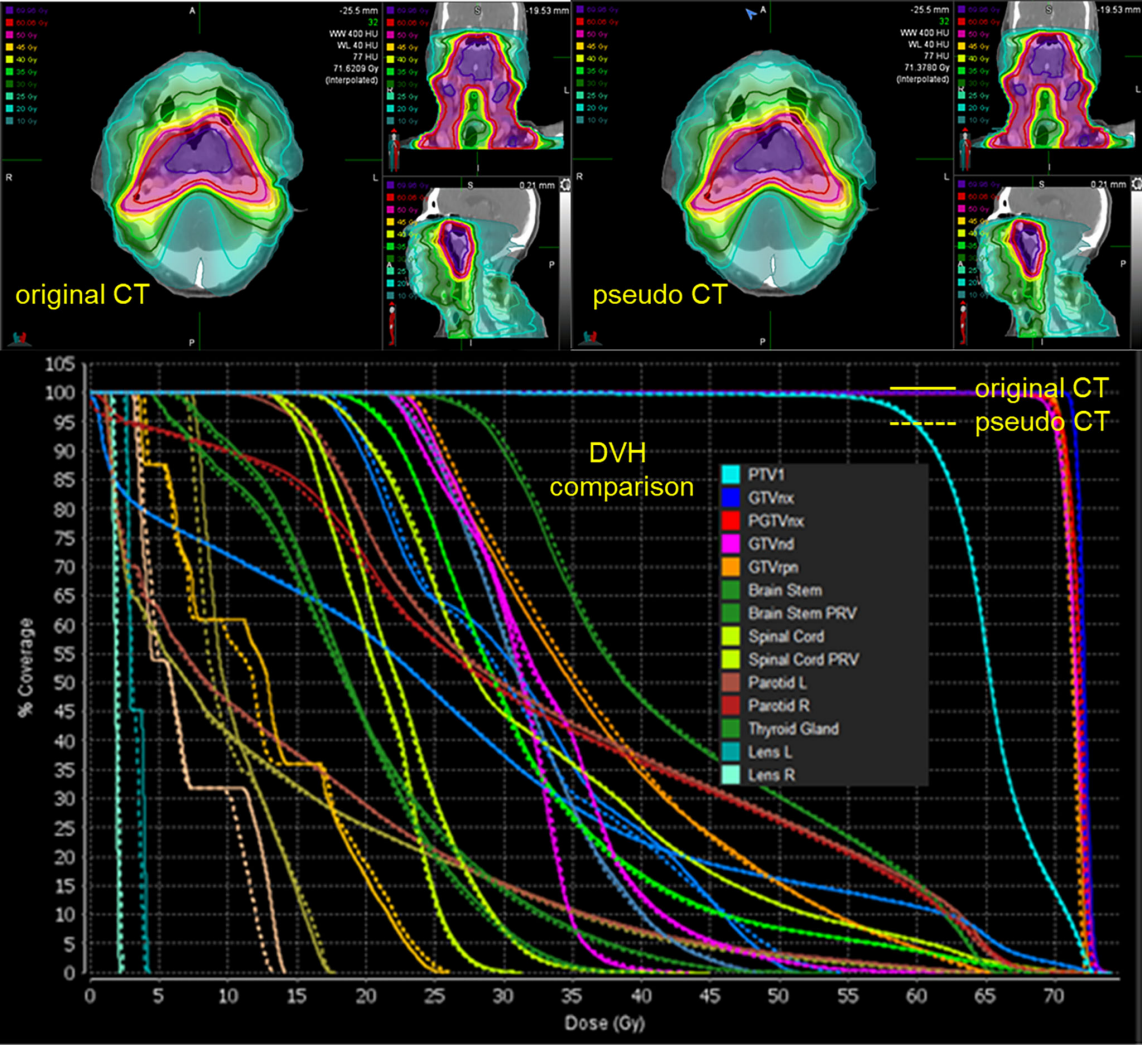
Spatial dose distributions of the original CT (left-up panel) and pCT (down-left panel) with identical beam assignment and their DVH comparison. The solid lines in the DVH correspond to the original CT, and the dotted lines correspond to pCT.

The spatial dose distribution of the pCT-based RT plan could be replicated very well on real CT with the same beam layout, and it demonstrates a good overlap between pCT and CT, regarding the structure DVH (**Figure 4**). Detailed DVH metric comparison is shown in **Table 3**.

**Table 3:** Reference dose values and dose uncertainties for dosimetry metrics.

Dosimetry metrics	PGTV D_99Gy_ (Gy)	PTV V_95%_ (%)	Lens D_max_ (Gy)
Reference value	69.73 ± 0.44	98.74 ± 0.39	4.22 ± 1.58
Dose uncertainty (relative value)	0.26 ± 0.10 (0.4% ± 0.2%)	0.1 ± 0.1	0.26 ± 0.20 (6.1% ± 4.6%)
**Dosimetry metrics**	**Spinal Cord D_max_ (Gy)**	**Brain Stem D_max_ (Gy)**	**Parotid V_30Gy_ (%)**
Reference value	32.20 ± 2.61	44.42 ± 6.48	52.76 ± 4.67
Dose uncertainty (relative value)	0.52 ± 0.51 (1.6% ± 1.5%)	0.68 ± 0.34 (1.6% ± 0.9%)	0.20 ± 0.17

**Figure 5:**
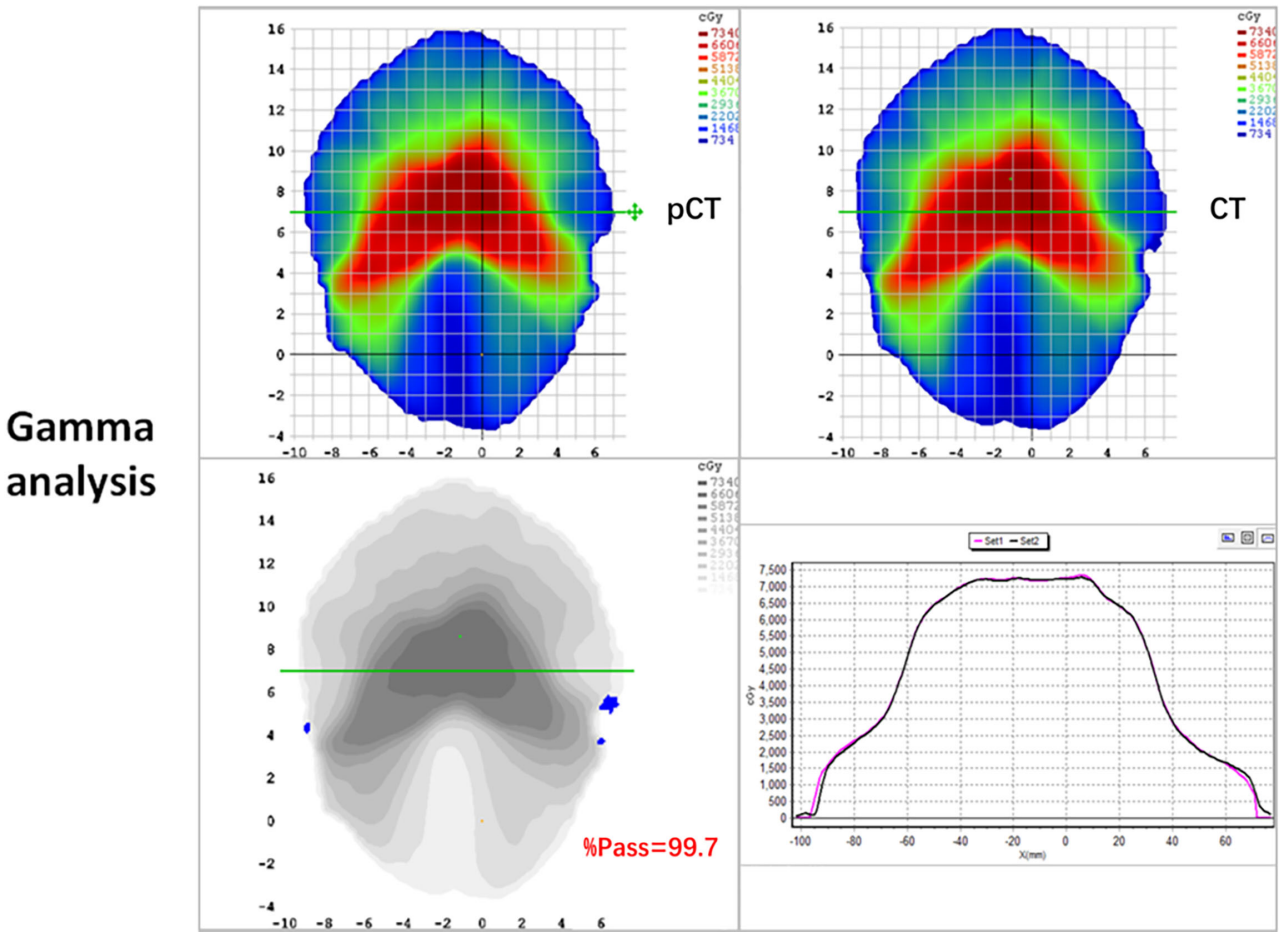
2D gamma analysis of pCT (up-left panel) and original CT dose distributions (up-right panel). The gamma pass rate of the slice is 99.7% (down-left panel), and the dose profiles are in good agreement in the high dose range (down-right panel).

The mean ( ± standard deviation) gamma pass rate of all the patients was 99.1% ± 0.3%, and the median gamma pass rate of all the selected slices was 99.3%, demonstrating a high consistency between the real CT and pCT-based RT planning. The worst slice pass rate was 95.9%, and the best was 100%. An exemplary gamma analysis result is shown in **Figure 5**. Notably, the positions that failed to pass the analysis are all in the peripheral areas because the CT and MR images were not acquired simultaneously, thus they could not be perfectly registered, especially near the outline.

## Discussion

In this study, deep CNN (U-Net) was used to generate pCT from T1 weighted MRI for NPC radiotherapy, and the dosimetric accuracy was assessed for pCT-based RT planning. We proved that the deep learning network can reliably convert MR images to pCT for HN position to provide ED information. Although deep learning network usually needs much training data, we achieved promising intensity and dosimetry prediction accuracy with limited data.

Previous studies on MR-pCT generation mostly focused on the brain or prostate, and only a few considered HN. The variance of existing brain pCT generation quality recorded by atlas-based studies is relatively large (average MAE from 85 to 184 HU) (3, 6, 19), which is attributed to the different data and image processing algorithms. For machine learning-based studies, Gudur et al. (20) used a Bayesian probability model and realized an average MAE of 126 HU for brain pCT generation. Despite the larger structure density variance around NPC than brain tumors, the pCT quality of the proposed model (MAE of 102.6 HU) is comparable, even superior to that in previous brain tumor studies. A direct comparison between the atlas and CNN-based pCT generation was performed by Han et al. (13), and the MAE of the CNN-based method was 10.26% lower than that of the atlas-based method, demonstrating the advantage of CNN-based pCT generation.

T1w MRI was used in this study, whereas a previous study on HN pCT generation (17) adopted T2w MRI for pCT generation using U-Net, and good pCT quality (MAE of 131 HU) was achieved. A dosimetric comparison was performed on a single patient regarding DVH metrics, and the difference between the minimum-dose-of-98%-volume (D98%) of high-risk, intermediate-risk, and low-risk PTVs on true CT and pCT was less than 1%. Herein, we further evaluated dosimetric accuracy in terms of global dose distribution consistency and OAR dose-volume metrics statistically. Combining our results and their study, regardless of the different network architecture, the performance of deep CNN on pCT generation from the two routine clinical MR modalities (i.e., T1w and T2w) prove to be promising, especially in clinical practice when it comes to MR-linac based adaptive RT, where both T1w and T2w MRI are possible to be adopted for online planning for each individual patient. Besides, although GAN is supposed to have a strong nonlinearity modeling ability (17), our results show that there is no significant difference in the performance of pix2pix GAN and U-Net. The training process of GAN can be improved *via* some sophisticated strategies, such as using other kinds of activation functions, cost functions, normalization, or optimizers, but this is beyond the scope of this study; thus, there is a need for further studies.

As for the computational efficiency of the proposed model, the average pCT generation time is 7.9 s using GPU acceleration, in contrast to several minutes or a few hours in the aforementioned atlas-based studies. The speed advantage of CNN is more important in MR-linac based radiotherapy, where online adaptive RT planning is needed.

For the key factors for CNN-based MRI-pCT generative model training, Andres et al. (21) evaluated the influence of training set size, MR sequence (T1w or contrast-enhanced T1w), MRI standardization approach, bias field correction, and architecture of CNN on the quality of brain pCT generation. They found that larger training set sizes result in higher pCT quality, whereas the other factors have no significant effect on the dosimetry quality, and all the candidate methods are relevant for potential use in clinical practice. The best MAE obtained using the slightly optimized preprocessing method was 78 ± 22 HU. Regarding the network architectures, 3%/3-mm gamma indices of 99.83% 0.19% and 99.74% 0.24% were obtained for HighRes-Net and 3D U-Net, respectively. Largent et al. (14) evaluated and compared U-Net and GAN using various loss functions (L2, single-scale perceptual loss (PL), multiscale PL, weighted multiscale PL), and patch-based method (PBM) based on T2w MRIs in prostate cancer. They found that GAN L2 and U-Net L2 show a lower MAE (≤34.4 HU) than U-Net PL, GAN PL, and PBM. The gamma pass rates were greater than 99% for all DLMs. GAN L2 and U-Net L2 provided the lowest dose uncertainties together with a low computation time. Their results show that the performance of U-Net and GAN is similar for pCT generation, which is consistent with our findings, although their study was for prostate pCT generation.

Besides conventional MRI modalities, such as T1w and T2w, other sequences were used to generate pCT. Many of the previous studies adopted the ultra-short time echo sequence, which could make the segmentation of bone and air easier, for brain pCT generation (22–26). However, MAEs in these studies ranged from 130 to 165 HU, which are not better than that of this study or the aforementioned conventional modality-based methods. Meanwhile, such dedicated MRI modalities result in additional scanning time, which is not conducive to their clinical applications.

The limitation of this study lies in the relatively low CT number and dose prediction accuracy at the surface of the patient. As mentioned in *Results* section, this may mainly due to the imperfect MR-CT registration and the residual MR distortions, despite the bias correction. A dedicated phantom experiment may be needed to further validate the performance of the proposed MR-pCT generative model, where a perfect MR-CT alignment could be implemented.

This study proved that deep CNN is an important tool to solve the problem of MRI to pCT generation for HN, with high conversion accuracy and efficiency. It can be of great value to the MRI-only radiotherapy community, especially those sites equipped with MR-linacs, by greatly reducing the additional imaging dose to patients and by ensuring the accuracy of delineation and dose calculation for each fraction.

## Data Availability Statement

The original contributions presented in the study are included in the article/supplementary material. Further inquiries can be directed to the corresponding authors.

## Ethics Statement

Ethical review and approval was not required for the study on human participants in accordance with the local legislation and institutional requirements. Written informed consent for participation was not required for this study in accordance with the national legislation and the institutional requirements.

## Author Contributions

Concept and design of study: XC, KM, and JD. Acquisition of data: XC and JL. Analysis and interpretation of data: XM and XC. Drafting the manuscript: XM and XC. Revising the manuscript critically for important intellectual content: XM, XC, YW, KM, and JD. All authors contributed to the article and approved the submitted version.

## Funding

This work was supported by the National Natural Science Foundation of China (11975313, 12005302), the Beijing Nova Program (Z201100006820058), the Beijing Municipal Science & Technology Commission (Z181100001918002), and the Beijing Hope Run Special Fund of Cancer Foundation of China (LC2019B06, LC2018A14).

## Conflict of Interest

The authors declare that the research was conducted in the absence of any commercial or financial relationships that could be construed as a potential conflict of interest.

## Publisher’s Note

All claims expressed in this article are solely those of the authors and do not necessarily represent those of their affiliated organizations, or those of the publisher, the editors and the reviewers. Any product that may be evaluated in this article, or claim that may be made by its manufacturer, is not guaranteed or endorsed by the publisher.
